# Non-Pungent *n*-3 Polyunsaturated Fatty Acid (PUFA)-Derived Capsaicin Analogues as Potential Functional Ingredients with Antioxidant and Carbohydrate-Hydrolysing Enzyme Inhibitory Activities

**DOI:** 10.3390/antiox8060162

**Published:** 2019-06-05

**Authors:** Mariarosaria Leporini, Monica Rosa Loizzo, Rosa Tundis, Chiara La Torre, Alessia Fazio, Pierluigi Plastina

**Affiliations:** Department of Pharmacy, Health Science and Nutrition, University of Calabria, Via Pietro Bucci, 87036 Arcavacata di Rende (CS), Italy; mariarosarialeporini@tiscali.it (M.L.); rosa.tundis@unical.it (R.T.); latorre.chiara@libero.it (C.L.T.); alessia.fazio@unical.it (A.F.)

**Keywords:** capsaicin, diabetes, fatty acid amides, PUFA, vanillylamides

## Abstract

*N*-Eicosapentaenoyl vanillylamine (EPVA) and *N*-docosahexaenoyl vanillylamine (DHVA), derived from *n*-3 polyunsaturated eicosapentaenoic acid (EPA) and docosahexaenoic acid (DHA), respectively, were studied for their potential antioxidant and carbohydrate-hydrolysing enzyme inhibitory activities together with capsaicin and the corresponding *n*-3 polyunsaturated fatty acids (PUFAs). The antioxidant potential was evaluated by 2,2-diphenyl-1-picrylhydrazyl (DPPH) assay, 2,2′-azino-bis(3-ethylbenzothiazoline-6-sulphonic acid (ABTS) assay, β-carotene bleaching test, and ferric reducing ability power (FRAP). In the ABTS assay the following trend of potency could be observed EPVA > DHVA ≥ capsaicin. In addition, except for the FRAP test, all samples showed a greater activity than the positive controls used as reference compounds in the antioxidant assays. Both EPVA and DHVA showed half maximal inhibitory concentration (IC_50_) values much lower than acarbose, which was used as the reference drug in the carbohydrate-hydrolysing enzyme inhibitory activity assays. It is interesting to note that structural changes in capsaicin derivatives had higher impacts on α-glucosidase than on α-amylase inhibition. Taken together, our data suggest that both EPVA and DHVA, which are not limited in compliance-related considerations with respect to capsaicin, due to absence of pungency, could be proposed as functional ingredients for the development of products for the management of type II diabetes and border-line hyperglycaemic patients.

## 1. Introduction

Diabetes mellitus (DM) is a chronic systemic disease in which hyperglycaemia, hyperinsulinemia, and hypertriglyceridemia are frequently associated [1]. The incidence of this disease, which now has 422 million patients, is set to rise dramatically in the coming years, particularly in regard to DM type 2 or T2DM (due to a progressive defect in secreting insulin on the background of insulin resistance) [2]. One of the most common actions to prevent T2DM is to promote lifestyle changes and reduce post-prandial hyperglycaemia [3]. To reduce the glycaemic peak, inhibitors of carbohydrate-hydrolysing enzymes, α-amylase and α-glucosidase, are frequently used [4]. Acarbose, one of the leading inhibitors of carbohydrate-hydrolysing enzymes, can cause several side effects [5]. Natural products can be used efficiently as inhibitors of these enzymes and often present lower side effects than acarbose [6]. In patients with T2DM, an insulin resistance condition prevents normal glucose uptake by muscle and fat tissue. This condition leads to elevated plasma glucose levels and promotes increases in the synthesis of radical oxygen species (ROS) [7]. For these reasons, in the last ten years there has been great interest in the research of both the hypoglycaemic and antioxidant properties of natural products and their derivatives.

The genus *Capsicum* represents a good source of dietary healthy compounds including ascorbic acid, carotenoids, flavonoids, phenolic acids, and tocopherols. These fruits are also rich in pungent capsaicinoids: capsaicin, dihydrocapsaicin, and related analogues [8]. All of these compounds are characterized by promising antioxidant activities by different mechanisms of action, including inhibition of iron and copper-mediated lipid peroxidation of low-density lipoproteins [9] and the reduction of metals that can act as hydrogen donors. Capsaicin has been reported to prevent oleic acid oxidation during cooking [10], as well as the autoxidation of linoleic acid [11]. In addition, capsaicin exerts other interesting healthy effects on inflammation, hyperalgesia, and peptic ulcers [12]. However, the pungency of capsaicin limits its use in food supplements or in nutraceutical products. Therefore, several non-pungent analogues were prepared and tested in order to obtain the same bioactivity without any type of limitations [13]. 

**Figure 1:**
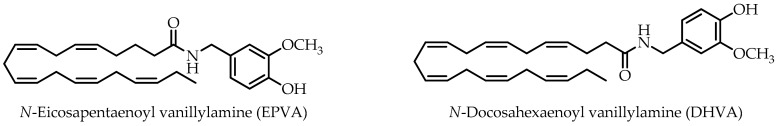
*n*-3 polyunsaturated fatty acid (PUFA)-derived capsaicin analogues.

As part of our research interest in the healthy properties of natural products from plant-based foods, spices, and their derivatives [14,15], the aim of this work was to explore the carbohydrate-hydrolysing enzyme inhibitory activities of capsaicin and its non-pungent derivatives *N*-eicosapentaenoyl vanillylamine (EPVA) and *N*-docosahexaenoyl vanillylamine (DHVA) (Figure 1). Their antioxidant potentials were investigated by 2,2-diphenyl-1-picrylhydrazyl (DPPH·) free radical scavenging assay, and their antioxidant capacities were determined by radical cation (ABTS·^+^) assay, β-carotene bleaching test, and the ferric reducing ability assay.

## 2. Materials and Methods 

### 2.1. Chemicals and Reagents 

Reagents were from Sigma-Aldrich (Milan, Italy), whereas analytical grade solvents were from VWR International (Milan, Italy). Acarbose (from *Actinoplanes* sp.) and Novozym®435 (Lipase B from *Candida antarctica*) were obtained from SERVA (Heidelberg, Germany) and Novozymes (Bagsværd, Denmark), respectively.

### 2.2. Chemistry

The synthesis of EPVA and DHVA was achieved as previously reported [16]. Briefly, eicosapentaenoic acid (EPA) or docosahexaenoic acid (DHA) was reacted with an equimolar amount of vanillylamine hydrochloride, in the presence of Novozym^®^435, an excess of trimethylamine, in 2-methyl-2-butanol. Reactions were carried out in an orbital shaker at 50 °C for 48 h. After filtration and evaporation of the solvent, purification of EPVA and DHVA was attained by column chromatography on silica gel (eluent: *n*-hexane-acetone). Their authenticity was assessed by comparing spectroscopic data with literature data [16], while purity (>98%) was evaluated by HPLC.

### 2.3. Carbohydrate-Hydrolysing Enzymes Inhibition

#### 2.3.1. α-Amylase Inhibitory Assay

The α-amylase inhibition assay was accomplished as previously described [17]. Absorbance was measured at 540 nm using a UV-Vis spectrophotometer (model V-550, Jasco, Milan, Italy). Acarbose was used as positive control. *Ki* values were calculated following the Cheng–Prusoff equation [18].

#### 2.3.2. α-Glucosidase Inhibitory Assay

The α-glucosidase inhibitory assay was achieved following the procedure previously described [17]. Absorbance was measured at 500 nm using a UV-Vis spectrophotometer (model V-550, Jasco, Milan, Italy). Acarbose was used as positive control. *Ki* value was calculated as previously described [18].

### 2.4. Investigation of Antioxidant Potential

#### 2.4.1. 2,2-Diphenyl-1-Picrylhydrazyl (DPPH) Free Radical Scavenging Assay

The DPPH assay was performed according to a previously reported protocol [19]. Absorbance was measured at 517 nm using a UV-Vis spectrophotometer (model V-550, Jasco, Milan, Italy). Ascorbic acid was used as positive control.

#### 2.4.2. ABTS, 2,2′-Azino-Bis(3-Ethylbenzothiazoline-6-Sulphonic Acid) Assay 

The ABTS assay was performed according to a previously reported protocol [20]. Absorbance was measured at 734 nm using a UV-Vis spectrophotometer (model V-550, Jasco, Milan, Italy) Ascorbic acid was used as positive control.

#### 2.4.3. β-Carotene Bleaching Test

The β-carotene bleaching test was performed as previously described [14]. The absorbance of the samples, standard, and control was measured using a UV-Vis spectrophotometer (model V-550, Jasco, Milan, Italy) at 470 nm against a blank at *t* = 0 and successively at 30 and 60 min. Propyl gallate was used as positive control.

#### 2.4.4. Ferric Reducing Ability Power (FRAP) Test

The FRAP test was performed following the protocol previously described [14]. Absorbance was measured using a UV-Vis spectrophotometer (model V-550, Jasco, Milan, Italy) at 470 nm. FRAP value was expressed as mM Fe(II)/g. Butylated hydroxytoluene (BHT) was used as a positive control.

### 2.5. Global Antioxidant Score (GAS)

The Global Antioxidant Score (GAS) is a statistical approach to evaluate the antioxidant potential of food samples subjected to different antioxidant assays. For its evaluation, the T-score was calculated by the following equation: T − score = (X − min)/(max − min), where min and max represent the smallest and largest values, respectively, of variable X among the investigated extract [21].

### 2.6. Statistical Analysis 

GraphPad Prism version 4.0 for Windows (GraphPad Software, San Diego, CA, USA) was used to calculate the concentration–response curve and consequently the half maximal inhibitory concentration (IC_50_). One-way ANOVA followed by a multicomparison Dunnett’s test (α = 0.05) was used to evaluate statistical differences (**** *p* < 0.0001, ** *p* < 0.05, * *p* < 0.1 compared with the positive controls). 

## 3. Results 

### 3.1. The Carbohydrate-Hydrolysing Enzyme Inhibitory Activities of Capsaicin Analogues

The potential carbohydrate-hydrolysing enzyme inhibitory properties of capsaicin and its PUFA-derived analogues were evaluated by measuring their effect on the activity of α-amylase and α-glucosidase. The results showed that capsaicin is more active than its derivatives (Table 1). In particular, IC_50_ values of 27.4, 32.8, and 31.2 µM were observed for capsaicin, EPVA, and DHVA, respectively, against α-amylase. In the α-glucosidase inhibition assay, similar IC_50_ values were found for EPVA and DHVA (23.6 and 23.9 µM, respectively), while capsaicin showed an IC_50_ value of 13.0 µM. In all cases, the IC_50_ values of capsaicin and its derivatives were much lower than the acarbose used as reference drug. Both derivatives showed *K*i values of 0.002 against all tested enzymes. No significant carbohydrate-hydrolysing enzymes inhibition was found for both EPA and DHA. 

### 3.2. Antioxidant Potential of Capsaicin Analogues

Different screening methods, namely DPPH test, ABTS test, β-carotene bleaching test, and FRAP assay, were used to investigate the potential antioxidant activity of capsaicin and the non-pungent PUFA-derivatives. Data are reported in Table 2.

A concentration-dependent trend was observed in all applied tests for all the samples, with the exception of the parent PUFAs. Although both DPPH and ABTS assays are used to study radical scavenging activity, the behaviour of the capsaicin derivatives against radicals looked different according to each of the assays. In fact, in the DPPH assay, EPVA and DHVA showed lower radical scavenging activities than capsaicin (IC_50_ values of 1.0, 0.8, and 0.6 µM, respectively). On the contrary, in the ABTS assay the following trends of potency could be observed EPVA > DHVA ≥ capsaicin.

In the β-carotene bleaching test, EPVA and capsaicin were the most active and did not differ significantly at both 30 and 60 min of incubation. On the contrary, the IC_50_ values of DHVA were much higher, indicating that this compound had lower bioactivity than its congeners.

**Figure 2:**
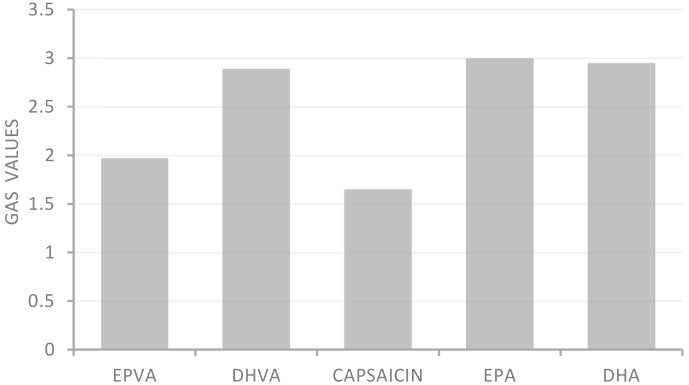
Global Antioxidant Score (GAS) values of *n*-3 PUFA-derived capsaicin analogues.

All samples showed moderate ferric reducing power, with FRAP values ranging from 18.2 to 30.0 μM Fe (II)/g. In addition, except for the FRAP test, all samples showed greater activity than the positive controls used as reference compounds in the antioxidant assays. Application of the statistical approach GAS evidenced the following trend: capsaicin > EPVA > DHVA > DHA > EPA (Figure 2).

## 4. Discussion

Several in vitro and in vivo studies evidenced that *Capsicum* extracts and their constituents are able to modulate glucose metabolism. Hypoglycaemic activities of lipophilic fractions of *C. annuum* var. *acuminatum* and var. *cerasiferum* have been reported against α-amylase, with IC_50_ values of 6.9 and 20.1 µg/mL, respectively [15]. IC_50_ values of 29.58 and 9.88 µg/mL were recorded for lipophilic fractions of both mature and immature fruits of Habanero pepper (*C. chinense*) against α-amylase [22], whereas the same extracts demonstrated a lower activity against α-glucosidase (IC_50_ of 265 and 150 µg/mL, respectively). A stronger α-amylase inhibitory activity was observed for big and medium *C. annuum* var. *acuminatum* (IC_50_ values of 8.7 and 29.0 µg/mL, respectively) [23]. Recently, the inhibitions of the carbohydrate-hydrolysing enzymes of two varieties of fresh Roggiano and Senise peppers have been reported [24]. These cultivars inhibited α-amylase with IC_50_ values of 68.9 and 55.3 µg/mL, respectively. A lower activity against α-glucosidase was observed for the same samples. A more recent investigation on the effects of a variety of chili peppers on carbohydrate-hydrolysing enzymes determined that all samples could inactivate both enzymes with different degrees of inhibition [25]. The highest anti-α-glucosidase and anti-α-amylase activities were found for “Sweet pepper” and “Green Chinda pepper”, respectively.

Capsaicin has been reported as the main component of peppers responsible for the observed bioactivities. It was able to decrease plasma glucose and to increase insulin levels in vivo [26]. In addition, an investigation of the hypoglycaemic activity (α-amylase and α-glucosidase inhibitory activity) of capsaicin using molecular docking and quantum calculation suggested that this compound has high potential to be developed as an alternative drug for diabetes [27]. In this context, the use of hybrid compounds derived from a food matrix has recently been proposed as a new potential means of managing hyperglycaemia [28]. In our work, we found that EPVA and DHVA, two non-pungent derivatives of capsaicin, have the ability to inhibit α-amylase and α-glucosidase at a level that is comparable to capsaicin. It is interesting to note that structural changes in capsaicin had a higher impact on α-glucosidase inhibition than on α-amylase. A great variability on the effect of capsaicin on insulin secretion was reported in literature as consequence of some parameters, such as way of administration and applied models (animal or humans). In vivo studies evidenced that administration of capsaicin and capsiate (orally at the dose of 6 mg/Kg for 28 days) inhibited intestinal glucose absorption in type 1 diabetic rats [29]. This study only partly confirmed previous results reporting the ability of the same pepper-derived compounds to improve glucose tolerance without affecting energy intake in diabetic rats [30]. Ahuja et al. demonstrated that capsaicin consumption is associated with increased insulin secretion [31]. On the contrary, plasma insulin concentrations measured after glucose loading in healthy human subjects following oral administration of capsaicin did not significantly differ from the control group [32]. Recently, we explored the effects of PUFA-derived capsaicin analogues EPVA and DHVA on insulin secretion in β-cells [16]. A significant increase in the release of insulin compared to the control was found after 15 and 120 min of incubation with EPVA. On the contrary, DHVA was ineffective at 15 min and induced a slight decrease of insulin secretion at 120 min. The action on insulin was mediated by an increase in the intracellular ATP/ADP ratio and a consequent Ca^2+^ influx through the voltage-gated channels. Notably, capsaicin was ineffective at both incubation times as well as EPA and DHA.

As previously mentioned, the burst of ROS as a consequence of high blood glucose levels is considered one of the key factors in the development of diabetes. The antioxidant capacity of medium and big *C. annuum* var. *acuminatum* has been investigated [23]. Big peppers showed a promising ABTS radical scavenging potential, with an IC_50_ of 16.4 µg/mL and the inhibition of linoleic acid oxidation (IC_50_ values of 1.2 and 2.9 µg/mL after 30 and 60 min of incubation). The bell pepper Roggiano exhibited the highest protection of lipid peroxidation IC_50_ values of 38.1 and 24.9 µg/mL for total extract and *n*-hexane fraction, respectively [24]. The mature fruits of Habanero pepper showed significant activity in the β-carotene bleaching test, with IC_50_ value of 4.57 µg/mL after 30 min of incubation [22]. In addition, Tundis et al. reported the results of ethanol extracts of two *C. annuum* cv *acuminatum* and *cerasiferum* [15]. The role of capsaicin as a free radical scavenger has been recently demonstrated [33]. Capsaicin efficiently reacts with oxygenated free radicals in aqueous solution. Different free radical mechanisms have been identified. For example, in the reaction of capsaicin with **^·^**OOH, ^·^OOCH_3_, and ^·^OCH_3_ single electron transfer is not predominant; in fact, for the peroxyl scavenging activity of capsaicin, hydrogen transfer from the hydroxyl of the phenolic group was more probable. For the reaction with methoxy radicals, by contrary, the hydrogen transfer from allylic sites are predicted to be the main channels of reaction. In our work, no significant differences were recorded among EPVA, DHVA, and capsaicin in terms of radical scavenging activity, regardless of the assay used. Differently, the presence of the DHA chain in DHVA derivative leaded to a decrease in the protection of lipid peroxidation with respect to both capsaicin and EPVA. This behaviour is in line with that reported by Cefarelli et al. who did not observe a correlation between the number of unsaturations (N.U.) and the protection of lipid peroxidation in the case of coumaryl fatty acid esters [34]. On the contrary, Zhou et al. found a positive correlation between N.U. and the protection of cupric ion-induced human low-density lipoprotein oxidation in the case of tyrosyl and hydroxytyrosyl esters [35]. These contradictory data could result from differences in applied methods, solubility, lipophilicity, and other parameters.

The Global Antioxidant Score (GAS), obtained as a combination of date from four antioxidant assays, allows the compound characterized by the highest antioxidant activity to be identified. From this approach, EPVA was observed to be more active than DHVA.

## 5. Conclusions

Non-pungent *n*-3 polyunsaturated fatty acid (PUFA)-derived capsaicin analogues were investigated for their potential antioxidant and carbohydrate-hydrolysing enzyme inhibitory activities together with capsaicin and the corresponding PUFAs. Results evidenced that the activities of EPVA and DHVA are not different from that of capsaicin, a compound considered useful in the treatment of the metabolic syndrome. Moreover, our new derivatives are characterized by the absence of spiciness, a feature that will make their use easier. Although the time for the clinical application of analogues of capsaicin in the treatment of metabolic disorders such as DM is still far away, our derivatives warrant further in vivo investigation.

## Figures and Tables

**Table 1:** Carbohydrate-hydrolysing enzyme inhibitory activities (IC_50_ µM).

Samples	α-Amylase	*Ki*	α-Glucosidase	*Ki*
EPVA	33 ± 2 ****	0.002	24 ± 2 ****	0.002
DHVA	31 ± 1 ****	0.002	24 ± 2 ****	0.002
Capsaicin	27 ± 1 ****	0.001	13 ± 1 ****	0.001
EPA	267 ± 9 ****	0.01	250 ± 9 ****	0.01
DHA	209 ± 9 ****	0.01	268 ± 9 ****	0.01
*Positive control*				
Acarbose	77 ± 2	0.004	54 ± 2	0.003

EPVA: *N*-eicosapentaenoyl vanillylamine; DHVA: *N*-docosahexaenoyl vanillylamine; EPA: eicosapentaenoic acid; DHA: docosahexaenoic acid. Data represent means ± S.D. (*n* = 3). Acarbose was used as positive control in α-amylase and α-glucosidase tests. Differences within and between groups were evaluated by one-way ANOVA followed by a multicomparison Dunnett’s test (α = 0.05): **** *p* < 0.0001 compared with the positive controls.

**Table 2:** Antioxidant activities of capsaicin and its derivatives.

Sample	DPPH Test IC_50_ (µM)	ABTS Test IC_50_ (µM)	β-Carotene Bleaching Test IC_50_ (µM)		FRAP Test (μM Fe(II)/g)
			30 min	60 min	
EPVA	1.0 ± 0.0 ****	0.2 ± 0.0 ****	9 ± 1 *	11 ± 1 *	28 ± 2
DHVA	0.8 ± 0.0 ****	0.3 ± 0.0 ****	46 ± 3 ****	56 ± 3 ****	18 ± 2
Capsaicin	0.6 ± 0.0 ****	0.3 ± 0.0 ****	10 ± 1 *	11 ± 1 *	30 ± 3
EPA	NA^^^	NA^^^	74 ± 5 ****	310 ± 10 ****	4.8 ± 0.8
DHA	NA^^^	NA^^^	70 ± 4 ****	287 ± 10 ****	9 ± 1
*Positive control*					
Ascorbic acid	23 ± 2	9.6 ± 0.9			
Propyl gallate			0.4 ± 0.0	0.4 ± 0.0	
BHT					63 ± 4

BHT: butylated hydroxytoluene. Data are expressed as means ± S.D. (*n* = 3). NA^^^: Not active at maximum concentration tested (0.125 μM). Differences within and between groups were evaluated by one-way ANOVA followed by a multicomparison Dunnett’s test (α = 0.05): **** *p* < 0.0001, * *p* < 0.1 compared with the positive controls.

## References

[1] Mathers C.D., Loncar D. (2006). Projections of global mortality and burden of disease from 2002 to 2030. PLoS Med..

[2] American Diabetes Association (2018). Classification and Diagnosis of Diabetes: Standards of Medical Care in Diabetes—2018. Diabetes Care.

[3] Blaslov K., Naranđa F.S., Kruljac I., Renar I.P. (2018). Treatment approach to type 2 diabetes: Past, present and future. World J. Diabetes.

[4] Alam F., Shafique Z., Amjad S.T., Bin Asad M.H.H. (2019). Enzymes inhibitors from natural sources with antidiabetic activity: A review. Phytother. Res..

[5] Shigeru K., Noboru N., Hisayuki S., Shigeyuki N. (1997). Comparison of the effects of acarbose and voglibose in healthy subjects. Clin. Ther..

[6] Ríos J.L., Francini F., Schinella G.R. (2015). Natural products for the treatment of Type 2 Diabetes Mellitus. Planta Med..

[7] Newsholme P., Cruzat V.F., Keane K.N., Carlessi R., de Bittencourt P.I. (2016). Molecular mechanisms of ROS production and oxidative stress in diabetes. Biochem J..

[8] Rosa A., Deiana M., Casu V., Paccagnini S., Appendino G., Ballero M., Dessí M.A. (2002). Antioxidant activity of capsinoids. J. Agric. Food Chem..

[9] Murakami K., Ito M., Htay H.H., Tsubouchi R., Yoshino M. (2001). Antioxidant effect of capsaicinoids on the metal-catalyzed lipid peroxidation. Biomed. Res..

[10] Henderson D.E., Henderson S.K. (1992). Thermal decomposition of capsaicin. 1. Interactions with oleic acid at high temperatures. J. Agric. Food Chem..

[11] Henderson D.E., Slickman A.M. (1999). Quantitative HPLC determination of the antioxidant activity of capsaicin on the formation of lipid hydroperoxides of linoleic acid: A comparative study against BHT and Melatonin. J. Agric. Food Chem..

[12] Srinivasan K. (2016). Biological activities of red pepper (*Capsicum annuum*) and its pungent principle capsaicin: A Review. Crit. Rev. Food Sci. Nutr..

[13] Panchal S.K., Bliss E., Brown L. (2018). Capsaicin in metabolic syndrome. Nutrients.

[14] Loizzo M.R., Pugliese A., Bonesi M., Tenuta M.C., Menichini F., Xiao J., Tundis R. (2016). Edible flowers: A rich source of phytochemicals with antioxidant and hypoglycemic properties. J. Agric. Food Chem..

[15] Tundis R., Loizzo M.R., Menichini F., Bonesi M., Conforti F., Statti G., De Luca D., De Cindio B., Menichini F. (2011). Comparative study on the chemical composition, antioxidant properties and hypoglycaemic activities of two *Capsicum annuum* L. cultivars (Acuminatum small and Cerasiferum). Plant Foods Hum. Nutr..

[16] Cione E., Plastina P., Perri M., Pingitore A., Caroleo M.C., Fazio A., Witkamp R., Meijerink J. (2019). Capsaicin analogues derived from *n*-3 polyunsaturated fatty acids (PUFAs) reduce inflammatory activity of macrophages and stimulate insulin secretion by β-cells in vitro. Nutrients.

[17] Loizzo M.R., Lucci P., Núñez O., Tundis R., Balzano M., Frega N.G., Conte L., Moret S., Filatova D., Moyano E. (2019). Native colombian fruits and their by-products: Phenolic profile, antioxidant activity and hypoglycaemic potential. Foods.

[18] Leff P., Dougall I.G. (1993). Further concerns over Cheng-Prusoff analysis. Trends Pharmacol. Sci..

[19] Fazio A., Caroleo M.C., Cione E., Plastina P. (2017). Novel acrylic polymers for food packaging: Synthesis and antioxidant properties. Food Pack. Shelf Life.

[20] Plastina P., Apriantini A., Meijerink J., Witkamp R., Gabriele B., Fazio A. (2018). In Vitro anti-inflammatory and radical scavenging properties of Chinotto (*Citrus myrtifolia* Raf.) essential oils. Nutrients.

[21] Todorovic V., Milenkovic M., Vidovic B., Todorovic Z., Sobajic S. (2017). Correlation between antimicrobial, antioxidant activity, and polyphenols of alkalized/nonalkalized cocoa powders. J. Food Sci..

[22] Menichini F., Tundis R., Bonesi M., Loizzo M.R., Conforti F., Statti G., De Cindio B., Houghton P.J., Menichini F. (2009). The influence of fruit ripening on the phytochemical content and biological activity of *Capsicum chinense* Jacq. cv Habanero. Food Chem..

[23] Tundis R., Loizzo M.R., Menichini F., Bonesi M., Conforti F., De Luca D., Menichini F. (2012). Air-dried capsicum annuum var. acuminatum medium and big: Determination of bioactive constituents, antioxidant activity and carbohydrate-hydrolyzing enzymes inhibition. Food Res Int..

[24] Loizzo M.R., Pugliese A., Bonesi M., De Luca D., O’Brien N., Menichini F., Tundis R. (2013). Influence of drying and cooking process on the phytochemical content, antioxidant and hypoglycaemic properties of two bell *Capsicum annum* L. cultivars. Food Chem. Toxicol..

[25] Watcharachaisoponsiri T., Sornchan P., Charoenkiatkul S., Suttisansanee U. (2016). The α-glucosidase and α-amylase inhibitory activity from different chili pepper extracts. Int. Food Res. J..

[26] Chaiyasit K., Khovidhunkit W., Wittayalertpanya S. (2009). Pharmacokinetic and the effect of capsaicin in *Capsicum frutescens* on decreasing plasma glucose level. J. Med. Assoc. Thai..

[27] Thongnum K., Chanthai S. (2018). Inhibitory reactivity of capsaicin with α-amylase and α-glucosidase related to antidiabetes using molecular docking and quantum calculation methods. Orient. J. Chem..

[28] Badolato M., Carullo G., Perri M., Cione E., Manetti F., Di Gioia M.L., Brizzi A., Caroleo M.C., Aiello F. (2017). Quercetin/oleic acid-based G-protein-coupled receptor 40 ligands as new insulin secretion modulators. Future Med. Chem..

[29] Zhang S., Ma X., Zhang L., Sun H., Liu X. (2017). Capsaicin reduces blood glucose by increasing insulin levels and glycogen content better than capsiate in streptozotocin-induced diabetic rats. J. Agric. Food Chem..

[30] Kwon D.Y., Kim Y.S., Ryu S.Y., Cha M.R., Yon G.H., Yang H.J., Kim M.J., Kang S., Park S. (2013). Capsiate improves glucose metabolism by improving insulin sensitivity better than capsaicin in diabetic rats. J. Nutr. Biochem..

[31] Ahuja K.D.K., Robertson I.K., Geraghty D.P., Ball M.J. (2006). Effects of chili consumption on postprandial glucose, insulin, and energy metabolism. Am. J. Clin. Nutr..

[32] Dömötör A., Szolcsányi J., Mózsik G. (2006). Capsaicin and glucose absorption and utilization in healthy human subjects. Eur. J. Pharmacol..

[33] Galano A., Martínez A. (2012). Capsaicin, a tasty free radical scavenger: mechanism of action and kinetics. J. Phys. Chem. B.

[34] Cefarelli G., D’Abrosca B., Fiorentino A., Izzo A., Monaco P. (2005). Isolation, characterization, and antioxidant activity of E- and Z-p-coumaryl fatty acid esters from cv Annurca apple fruits. J. Agric. Food Chem..

[35] Zhou D.-Y., Sun Y.-X., Shahidi F. (2017). Preparation and antioxidant activity of tyrosol and hydroxytyrosol esters. J. Funct. Foods.

